# T242. DEVELOPMENT OF SELF-STIGMA INVENTORY FOR PATIENTS WITH SCHIZOPHRENIA (SSI-P): RELIABILITY AND VALIDITY STUDY IN TURKEY

**DOI:** 10.1093/schbul/sby016.518

**Published:** 2018-04-01

**Authors:** Mustafa Yildiz, Fatma Kiras, Aysel İncedere, Fatma Betül Abut

**Affiliations:** Kocaeli Universitesi

## Abstract

**Background:**

Stigmatization is defined as discrimination and loss of social status which is triggered by negative stereotypes related to certain human characteristics such as having mental illnesses. Most of the people with mental illnesses are aware of the stigmatization in society and some of them internalize this social stigma by stigmatizing themselves. Schizophrenia is known as the most stigmatized mental illness by the society, healthcare professionals, and the patients themselves. In Turkey, there is no scale that evaluates the self-stigmatization of people with schizophrenia. The purpose of this study was to develop a culturally-sensitive and easy-to-use instrument to measure self-stigma of the people with schizophrenia.

**Methods:**

After examining the existing stigma and self-stigma scales for people with mental illnesses, 25-item self-stigma inventory was formed. Focus group interviews were conducted with 20 patients with schizophrenia and the items of the newly developed form were reviewed and rephrased into more comprehensible statements for the patients. The pilot study was conducted with a sample of 15 patients with schizophrenia and the inventory was finalized as 19-item self-stigma inventory for the patients. One hundred and sixty-two outpatients with schizophrenia or schizoaffective disorder were given sociodemographic form, Self-Stigma Inventory (SSI-P), Beck Depression Inventory (BDI), Internalized Stigma of Mental Illness (ISMI) Scale, Rosenberg Self-Esteem Scale (RSES), Beck Hopelessness Scale (BHS), Positive and Negative Syndrome Scale (PANSS), Clinical Global Impression-Severity (CGI-S), and Global Assessment of Functioning (GAF). For reliability analyses; split-half reliability, internal consistency coefficient, and item-total correlation were assessed. For validity analyses; explanatory factor analysis and convergent validity were conducted.

**Results:**

The sample of the study was 162 outpatients. Seventy-seven percent of the participants were males, 70% were single, mean age was 37, and level of education was 10 years. Cronbach’s alpha coefficient for SSI-P total score was 0.93, and Cronbach’s alpha scores for SSI-P subscales were between 0.60 and 0.91. Split-half reliability of the inventory was 0.90. For factor analysis, Kaiser-Meyer-Olkin value was found as 0.913 and Barlett test was significant (p<0.001). In explanatory factor analysis, three factors (perceived incompetency, internalized stereotypes and social withdrawal, and concealment of the illness) were defined and 63% of the variance was explained by the factors. Two items were removed from the questionnaire as they had lower item value than 0.40. In the final form, perceived incompetency factor consisted of 8 items, internalized stereotypes and social withdrawal factor had 7 items, and concealment of the illness factor had 2 items. SSI-P total score was found significantly and positively correlated with PANSS negative symptoms subscale (r=0.19, p<0.05), Beck Depression Inventory (r=0.53, p<0.001), Beck Hopelessness Scale (r=0.40, p<0.001), ISMI total score (r=0.73, p<0.001), and Rosenberg Self-Esteem Scale (r=-0.59, p<0.001).

**Discussion:**

The results of the current study show that SSI-P is a reliable and valid instrument for assessing the self-stigmatization of the patients with schizophrenia. It consists of 17 items that are comprehensible and user-friendly for the patients. The scale could be considered as an important instrument in psychotherapy practices and for research purposes.

